# The role of non-coding RNAs in muscle aging: regulatory mechanisms and therapeutic potential

**DOI:** 10.3389/fmolb.2023.1308274

**Published:** 2024-01-09

**Authors:** Yeo Jin Shin, Ki-Sun Kwon, Yousin Suh, Kwang-Pyo Lee

**Affiliations:** ^1^ Aging Convergence Research Center, Korea Research Institute of Bioscience and Biotechnology (KRIBB), Daejeon, Republic of Korea; ^2^ Department of Bioscience, KRIBB School, Korea University of Science and Technology (UST), Daejeon, Republic of Korea; ^3^ Aventi Inc., Daejeon, Republic of Korea; ^4^ Department of Obstetrics and Gynecology, Columbia University, New York, NY, United States; ^5^ Department of Genetics and Development, Columbia University, New York, NY, United States

**Keywords:** ncRNA, miRNA, lncRNA, circRNA, skeletal muscle, aging

## Abstract

Muscle aging is a complex physiological process that leads to the progressive decline in muscle mass and function, contributing to debilitating conditions in the elderly such as sarcopenia. In recent years, non-coding RNAs (ncRNAs) have been increasingly recognized as major regulators of muscle aging and related cellular processes. Here, we comprehensively review the emerging role of ncRNAs, including microRNAs (miRNAs), long non-coding RNAs (lncRNAs), and circular RNAs (circRNAs), in the regulation of muscle aging. We also discuss how targeting these ncRNAs can be explored for the development of novel interventions to combat age-related muscle decline. The insights provided in this review offer a promising avenue for future research and therapeutic strategies aimed at improving muscle health during aging.

## 1 Introduction

Muscle aging refers to progressive decline in muscle mass, strength, and function that occurs as individuals grow older (Akasaki et al., 2014; Bai et al., 2016). This age-related phenomenon is characterized by various physiological changes in skeletal muscle tissue, including alterations in muscle fiber composition, reduced muscle protein synthesis, and impaired muscle regeneration (Fry and Rasmussen, 2011; Lee et al., 2022; Ato et al., 2023). Understanding the mechanisms and factors involved in muscle aging is crucial for developing strategies to mitigate its effects and improve the quality of life for older individuals.

Non-coding RNAs (ncRNAs) are a varied family of RNA that do not code for proteins but are crucial for many biological activities, including gene regulation, epigenetic modifications, and chromatin remodeling (Mattick et al., 2023). This class of RNAs includes microRNAs (miRNAs), long non-coding RNAs (lncRNAs), circular RNAs (circRNAs), and others. Non-coding RNAs have emerged as critical players in the regulation of various cellular processes, including those governing muscle tissue (Mori et al., 2019). In the context of muscle aging, research has uncovered a wealth of information about the roles ncRNAs play in mediating muscle loss, muscle regeneration, and overall muscle maintenance. For instance, modulating these miRNAs, such as miR-29, miR-143, and miR-431, could potentially improve age-related muscle regeneration (Hu et al., 2014; Lee et al., 2015; Soriano-Arroquia et al., 2016a).

We aim to provide a comprehensive and up-to-date overview of miRNAs, lncRNAs, and circRNAs, summarizing research conducted over the past 5 years. We delve into the specific roles of these ncRNAs in modulating muscle aging process. By shedding light on the dynamic interplay between non-coding RNAs and muscle aging, this review aims to contribute to our understanding of the molecular underpinnings of sarcopenia and to pave the way for innovative therapeutic strategies that may 1 day counteract the debilitating effects of muscle aging.

## 2 Regulatory mechanisms of non-coding RNAs in muscle aging

### 2.1 miRNAs in muscle aging

MicroRNAs are short non-coding RNA molecules, typically 21–23 nucleotides in length, that play a vital role in fine-tuning gene regulation across various organisms (Ambros, 2004). They originate as primary miRNA transcripts transcribed by RNA pol II or III (Bartel, 2004; Shang et al., 2023). Subsequently, precursor miRNAs, which are produced by the Drosha complex from pri-miRNAs, are then further refined by Dicer to create mature miRNAs in the cytoplasm. Mature miRNAs direct the RNA-induced silencing complex (RISC) to target mRNAs, resulting in translational repression or mRNA degradation in various physiological conditions. Numerous studies have revealed that the miRNA-mRNA regulatory network in skeletal muscle undergoes changes with age (Mercken et al., 2013; Kim et al., 2014; Sun et al., 2015; Soriano-Arroquia et al., 2016b).

The investigation into stem cell-based therapies as a promising strategy to combat muscle aging and rejuvenate skeletal muscle tissue has gained significant attention (Neves et al., 2017; Evano and Tajbakhsh, 2018). Moreover, recent research has shed light on the role of miRNAs in this context, linking their functions to muscle regeneration and the aging skeletal muscle. In 2016, miR-501-3p was identified as a muscle-specific miRNA enriched in activated myogenic progenitor cells during muscle regeneration (Fahrner et al., 2023). Subsequent research demonstrated that miR-501 knockout mice exhibited a significant reduction in the diameter of newly formed myofibers. This result is a result of miR-501 controlling the expression of the sarcomeric gene via the estrogen-related receptor gamma (Esrrg). Another noteworthy miRNA, miR-7a-1, has been identified as highly expressed in aged muscle and as a downstream factor of HuR and Msi2 (Yang et al., 2022). This miRNA plays a role in inhibiting the translation of Cry2 and modulating MuSC (Muscle Stem cell)s differentiation. These findings contribute to our understanding of how miRNAs are involved in muscle regeneration and the aging process.

Cellular senescence, characterized by irreversible growth arrest, is a hallmark of stem cell aging (Munoz-Espin and Serrano, 2014). Cellular senescence also can lead to various age-related muscle changes, impaired muscle regeneration, and increased susceptibility to muscle injuries (He et al., 2021). Some miRNAs, known as senescence-associated miRNAs, are identified that differentially expressed during senescence contribute to its establishment and maintenance (Munk et al., 2017; Panda et al., 2017). For instance, miR-24 has been found to be downregulated in FACS-sorted MuSCs and regenerating muscle during aging (Soriano-Arroquia et al., 2021). miR-24 regulates the generation of mitochondrial ROS through Prxd6 and subsequently influences MuSCs viability, myogenic potential and senescence. Modulating miR-24 in aged mouse are preserve satellite cells viability and mitochondria function. Another study reveals that replicative senescence suppresses the expression of muscle-specific miRNAs in myoblasts. In particular, expression of miR-1 and miR-133a were suppressed in late passage C2C12 myoblasts (Shintani-Ishida et al., 2023). Consistently with these findings, the expression of miR-1 and miR-133a were downregulated in primary myoblasts isolated from aged muscle. Senescent myoblasts exhibit inhibited myogenic differentiation, but overexpression of miR-1 or miR-133a leads to improved cell fusion rates.

In recent research, the potential of EGCG (Epigallocatechin gallate) supplementation, a notable polyphenol demonstrated the ability to elevate the expression of miRNA-486-5p in both aging SAMP8 mice (Senescence-Accelerated Mouse Prone 8), and in C2C12 cells (Chang et al., 2020). In aged mice and cells, EGCG increases miR-486, which inhibits FoxO1a-mediated MuRF1 (Muscle RING Finger 1) and Atrogin-1 (Muscle Atrophy F-box) transcription through activating AKT phosphorylation. Moreover, it was observed that late passage C2C12 cells exhibited increased myostatin expression, and the application of anti-myostatin treatment led to an upregulation in the expression of miR-486-5p. Collectively, these studies shed light on the intricate role of miRNAs in stem cell aging and muscle senescence, highlighting their potential as therapeutic targets to counteract the effects of aging on muscle tissue.

Sarcopenia, characterized by age-related muscle loss, is influenced by various factors, with increased expression of E3 ligases like MuRF1 and Atrogin-1 in aged muscles, highlighting their involvement in the ubiquitin-proteasome (Kitajima et al., 2020). Recent research has identified specific miRNAs associated with sarcopenia that target or modulate these E3 ligases, underscoring their importance in maintaining muscle. Notably, miRNAs located within the *Dlk1-Dio3* cluster have induced hypertrophic phenotypes in C2C12 myotubes (Shin et al., 2020). Among these miRNAs, including miR-376c, miR-668, miR-1197, miR-495, miR-377, miR-379, and miR-431, they directly bind to the 3′UTR of Atrogin-1, leading to the suppression of Atrogin-1 in both human and mouse muscle cells. Furthermore, miR-376c has shown remarkable potential in ameliorating skeletal muscle atrophy and improving muscle function in old mice. These miRNAs consistently exhibit downregulation in aged human muscles.

Recent studies have reported on the regulation of mitochondrial homeostasis controlling muscle mass (Short et al., 2005; Yeo et al., 2019). It was shown that miR-181a is crucial in controlling the age-related alteration of mitochondrial dynamics in muscle via targeting p62 and Park2 (Goljanek-Whysall et al., 2020). *In vivo* restoration of miR-181a levels in the muscles of old mice inhibited the accumulation of p62, Park2, and DJ-1 while maintaining mitochondrial content. In the end, this enhanced the size and force of myofibers. Collectively, these results indicate that miR-181a functions as an effective mitochondrial dynamics regulator, both *in vitro* and *in vivo*. Liang and others conducted a study that revealed changes in miRNA expression patterns in aged rat muscle (Liang et al., 2023). They observed upregulation of miR-99a-5p, miR-7a-1-3p, miR-28-5p, miR-151-5p, miR-135a-5p, and miR-196b-5p, while miR-136-5p, miR-127-3p, miR-206-3p, miR-674-5p, miR-222-3p, miR-652-3p, and miR-708-5p were downregulated. Notably, among the upregulated miR-151-5p, miR-135a-5p, miRNAs, miR-7a-1-3p, and miR-196b-5p were found to target genes associated with muscle atrophy, including Hif1a, Ar, Fak, Igf1, Bdnf, and Nras. These identified miRNAs have the potential to regulate age-related muscle atrophy, suggesting their involvement in the development of muscular atrophy associated with aging.

#### 2.1.1 Extracellular miRNAs in muscle aging

Extracellular miRNAs refer to microRNAs that are found outside of cells. These miRNAs can be detected in the bloodstream and other extracellular spaces, and they play a role in regulating gene expression and cellular processes related to physiology, including muscle aging (Mori et al., 2019). A recent study has provided insights into this phenomenon by examining exosome vesicle (EV) markers, CD9 and CD81, which decrease with the aging process (Xhuti et al., 2023). *In vitro* experiments involved inducing senescence in human skeletal myotubes, serving as an *in vitro* model of aging. In this model, secreted EV markers were observed to significantly decrease. Notably, late-passage myotubes exhibited abnormal production of endogenous myomiRs (miR-1, miR-133a, miR-206), which were less abundant compared to their youthful counterparts. Yedigaryan et al. (2022) investigated extracellular vesicles obtained from animal models of both muscle atrophy and hypertrophy brought on by aging and chronic disease. Their comprehensive analysis delved into the transcriptome, protein contents, and miRNAs, leading to the identification of a promising anti-atrophic miRNA signature, notably featuring miR-1 and miR-208a. To validate its potential, they conducted experiments using mesoangioblasts (MABs), adult stem cells linked to blood vessels, and observed a substantial improvement in myogenic differentiation efficiency upon exposure to this miRNA signature. Moreover, when miRNA-treated MABs were injected into aged muscle tissue, they observed significant enhancements in various skeletal muscle parameters, including muscle fibrosis, cross-sectional area, muscle weight, and strength, compared to control groups. Fukuoka et al. (2021) revealed changes in cell free-miRNA(cf-miRNA)s between young and old mouse blood. MiR-199-3p, one of these miRNAs, was found to be much lower in old blood compared to young blood. Since miR-199-3p can enhance muscle differentiation and regeneration, giving it to old mice led to the enlargement of the muscle fibers. These findings highlight the substantial influence of aging on skeletal muscle, primarily through alterations in EV or cf-miRNAs.

Extracellular miRNAs, functioning as messengers, play a pivotal role in facilitating communication between organs (Ji and Guo, 2019). Recent reports provide compelling evidence of intercellular interactions between myofibers and MuSCs within the context of muscle aging. Specifically, miR-690, originating from atrophic muscle fibers, inhibit MuSCs myogenic capacity by targeting MEF2 during aging (Shao et al., 2022). Our knowledge of the quantity of MuSCs and their ability for myogenesis during age-induced muscle atrophy is improved by EV-mediated intercellular communication between muscle fibers and MuSCs. Furthermore, by targeting miR-690, it may be possible to enhance muscle mass and strength, ultimately addressing the challenges posed by sarcopenia in the aging population. Fulzele et al. (2019) have indicated that EVs derived from muscle tissue, specifically those positive for α-sarcoglycan, exhibit a significant increase in miR-34a levels with age. These miR-34a-enriched EVs also accumulate in response to oxidative stress induced in C2C12 myotubes and primary human myotubes *in vitro*. Importantly, these EVs have been found to reduce the viability of bone marrow mesenchymal stromal cells (BMSCs) and promote BMSC senescence both *in vitro* and *in vivo*. These results imply that circulating senescence-associated EVs may originate from old skeletal muscle and may have direct effects on stem cell populations in tissues like bone through the transfer of their microRNA cargo. Another study has demonstrated that transplantation of exosomes derived from perimuscular adipose tissue (PMAT) to limb muscles results in a reduction in the number of α7/CD29-double positive myogenic progenitor cells (Itokazu et al., 2022). Functional analysis has unveiled that let-7d-3p, present in exosomes from aged PMAT, regulates these cells by targeting HMGA2, a transcription factor crucial for stem cell self-renewal. In the context of age-associated muscular atrophy, these findings suggest a novel way of interaction between adipose tissue and skeletal muscle and suggest that adipose tissue-derived miRNAs may be crucial in the development of sarcopenia. Understanding these miRNA-mediated interactions contributes to a comprehensive insight of the physiological changes associated with aging and may hold promise for developing interventions that target multiple organ systems, promoting healthy aging, and mitigating age-related muscle decline.

MiRNA has the potential to be utilized as a predictive biomarker for assessing the risk of developing sarcopenia and as a diagnostic marker to confirm the presence of muscle aging. Recent research has also focused on the discovery of new miRNAs for diagnosing sarcopenia. To establish a biomarker reflecting age-related loss of muscle quantity and quality, Liu et al., 2021a conducted a comparative analysis of serum samples obtained from 77 elderly participants (aged 66–92 years), who were divided into three groups (normal, dynapenia, sarcopenia). They identified that miR-486 was downregulated in both dynapenia and sarcopenia, while miR-146a was specifically downregulated in sarcopenia. The AUC values for miR-486 and miR-146a were 0.708 and 0.676, respectively. Furthermore, sarcopenic older adults exhibited lower levels of circulating miR-146a, along with reduced handgrip strength, suggesting miR-146a may serve as a potential marker for sarcopenia. Another group of researchers reported that plasma levels of miR-155, miR-208b, miR-499, miR-328, miR-222, and miR-210 decrease in response to sarcopenia in elderly individuals (He et al., 2020). Similar to the miR-146a study, miR-208b and miR-155 showed significant correlations with handgrip strength in women, while changes in miR-499, miR-208b, and miR-222 were significantly correlated with Appendicular Skeletal Muscle Mass Index (ASMI) in men when comparing the sarcopenia group to the non-sarcopenia group.

Sarcopenia not only involves a decrease in muscle function but also its correlation with various diseases. Indeed, research findings have explored the associations of sarcopenia, extending beyond its diagnostic aspects, with other diseases. Patients with Congestive Heart Failure (CHF) reported that lower hand-grip strength, ASMI and physical performance than healthy human. In CHF patients, there is a notable upregulation of circulating miR-21 and downregulation of miR-133a, miR-181a, miR-434-3p, and miR-455-3p when compared to healthy controls (Qaisar et al., 2021). Among these miRNAs, it has been previously reported that miR-434-3p and miR-455-3p are downregulated in the gastrocnemius (GA) muscle of aged mice (Kim et al., 2014), plasma from disuse-induced atrophy models (Jung et al., 2017) and miR-434-3p has also been implicated in mechanically regulating age-associated apoptosis through eIF5A1 in skeletal muscle (Pardo et al., 2017). Hence, there is potential for miR-455-3p and miR-434-3p to serve as a biomarker for both CHF and sarcopenia. Wang and others investigated the connection between glioma and muscle aging with the goal of glioma prognosis using co-expressed genes (Wang et al., 2022). They discovered seven survival-related genes, including FOSL1, KRAS, STAT3, ALCAM, ERBB2, IFNB1, and EN2, as well as eight co-downregulated miRNAs, three co-upregulated miRNAs, and three co-regulated miRNAs. By adding FOSL1 and EN2 into a multi-factor Cox regression model, they were able to produce ROC curves with AUC values of 0.702 and 0.709, suggesting these genes can predict glioma prognosis. Furthermore, by applying Gene Set Enrichment Analysis, they identified miR-33a as a regulator targeting both FOSL1 and EN2. This indicates mir-33a as a promising predictor for glioblastoma and muscle reduction. These miRNA-based biomarkers have the potential to play a pivotal role in facilitating early intervention strategies and enabling the development of personalized treatment approaches. These findings make it possible to consider the application of miRNAs as biomarkers for diagnosing sarcopenia. These miRNAs characterized in muscle aging were summarized in Table 1.

**TABLE 1:** Characterization of miRNAs in muscle aging.

miRNAs	Expression^a^	Target genes	Biological function	Reference
miR-501-3p	↓	Esrrg	miR-501 KO mice exhibit a reduction of muscle regeneration	Fahrner et al. (2023)
miR-7a-1	↑	Cry2	Inhibition of MuSCs differentiation	Yang et al. (2022)
miR-24	↓	Prdx6	Overexpression of miR-24 are preserved MuSC viability and mitochondrial fucntion	Soriano-Arroquia et al. (2021)
miR-1 and miR-133a	↓	?	miR-1 and miR-133a leads to improved cell fusion rates	Shintani-Ishida et al. (2023)
miR-486-5p	↓	?	miR-486-5p activates phosphorylation of AKT, inhibiting FoxO1a-mediated MuRF1 and Atrogin-1	Chang et al. (2020)
miR-668, miR-376c, miR-1197, miR-495, miR-377, miR-379 and miR-431	↓	Atrogin-1	These miRNAs exhibit anti-atrophic phenotypes *in vitro* and miR-376c ameliorating skeletal muscle atrophy	Shin et al. (2020)
miR-181a	↓	p62 and Park2	Overexpression of miR-181a increases myofiber size and enhances muscle force	Goljanek-Whysall et al. (2020)
miR-7a-3p, miR-135a-5p, miR-151-5p and miR-196b-5p	↑	Ar, Igf1, Hif1a, Bdnf, Fak and Nras	Activation of age-related muscle atrophy	Liang et al. (2023)
miR-1 and miR-208a	↓	?	Treatment of miRNAs ameliorates in muscle strength, Cross Section Area, fibrosis	Yedigaryan et al. (2022)
miR-199-3p	↓	?	Enhanced aged muscle regeneration and improved muscle strength in mdx mice	Fukuoka et al. (2021)
miR-690	↑	Mef2	Inhibited MuSCs myogenic capacity	Shao et al. (2022)
miR-34a	↑	?	Muscle derived miR-34a promotes BMSC senescence	Fulzele et al. (2019)
Let-7d-3p	↑	HMGA2	PMAT derived let-7d-3p blocks muscular stem cell proliferation in aged muscle	Itokazu et al. (2022)
miR-146a	↓	?	Circulating miR-146a correlated with handgrip strength in sarcopenia patients	Liu et al. (2021a)
miR-208b and miR-155	↓	?	These miRNAs correlated with handgrip strength in sarcopenic woman	He et al. (2020)
miR-208b, miR-499 and miR-222	↓	?	These miRNAs correlated with ASMI in sarcopenic man	He et al. (2020)
miR-21	↑	?	miR-21 is upregulated in both CHF and Sarcopenia patients	Qaisar et al. (2021)
miR-181a, miR-133a, miR-434-3p, miR-455-3p	↓	?	These miRNAs are downregulated in both CHF and Sarcopneia patients	Qaisar et al. (2021)
miR-33a	↓	FOSL1 and EN2	miR-33a has a good predictive value for glioblastoma and skeletal muscle reduction	Wang et al. (2022)

^a^ The arrows indicating the expression of miRNAs, in aged muscle or serum, comparing to young control, was as follows.

### 2.2 lncRNAs in muscle aging

Long non-coding RNAs, typically consisting of over 200 nucleotides, do not encode proteins but perform various crucial functions within the cell (Okazaki et al., 2002; Mattick, 2009). Many lncRNAs undergo processes such as splicing and polyadenylation, similar to coding mRNAs (Statello et al., 2021). The addition of a polyadenylation tail to lncRNAs serves several purposes, including protecting them from degradation, facilitating their transport from the nucleus to the cytoplasm, and influencing their interactions with other cellular molecules, including RNA-binding proteins and miRNAs. Additionally, lncRNAs function as scaffolds, decoys, and enhancers, participating in intricate molecular interactions that affect nuclear organization (Mattick et al., 2023). Through these diverse mechanisms, lncRNAs play roles in gene expression regulation, chromatin structure modulation, and the modulation of cellular signaling pathways.

Over the years, many researchers have unveiled the intricate molecular mechanisms underpinning age-related muscle atrophy, with ncRNAs assuming a central role in this complex process. Zheng et al. (2021) created a co-expression network between these lncRNAs and mRNAs in a study that compared lncRNA expression profiles in skeletal muscle biopsies from old and young subjects. Their findings revealed that lncRNAs PRKG1-AS1, AC004797.1, and GRPC5D-AS1 might play a role in aging-related muscle atrophy. Notably, functional investigation showed that the expression of MyoD, MyoG, and Mef2c could be increased by knockdown of PRKG1-AS1. Synaptopodin-2 (SYNPO2) intron sense-overlapping lncRNA, known as SYISL, has homologues in humans and pigs, and it regulates myogenesis through its interaction with enhancer of zeste homologue 2 (EZH2) (Jin et al., 2018). Recent studies have reported that a gradual upregulation of SYISL during the aging process in mice (Jin et al., 2022). This lncRNA also plays a significant role in muscle atrophy and sarcopenia. It is upregulated during muscle atrophy, promotes muscle atrophy in Dexamethasone-induced muscle atrophy models, and can alleviate muscle atrophy and sarcopenia when knocked down or knocked out in mice. Mechanistically, SYISL increases the expression of muscle atrophy-related genes, such as FOXO3a, Atrogin-1 and MuRF1, by acting as a sponge for miR-103-3p/miR-23a-3p/miR-205-5p, thereby contributing to its role in maintaining muscle homeostasis. Li et al. reported that LncMAAT expression is reduced not only in muscle aging but also in various types of muscle atrophy, including AngII infusion, fasting, and immobilization (Li et al., 2021). Overexpression of lncMAAT demonstrated the ability to alleviate muscle atrophy *in vitro* suppressed Atrogin-1 and MuRF1. Mechanistically, the neighboring gene Mbnl1’s expression is favorably regulated by lncMAAT via a cis-regulatory module, and miR-29b’s transcription is negatively regulated by lncMAAT through SOX6. Timmons et al. established that an innovative method for quantifying coding and long non-coding RNA transcripts in 577 adult human muscle and brain samples using variation in protein-coding transcript expression and linear modeling (Timmons et al., 2019). Remarkably, they are identified approximately 800 transcripts associated with age up to around 60 years. *In silico* analysis showed that this age-related transcript signature was influenced by drugs affecting longevity, particularly those targeting the mTOR pathway. The study also highlighted the importance of lncRNAs identified specific genes, such as ECSIT, UNC13, and SKAP2, linked to age-related processes and mitochondrial gene expression, highlights on potential factors influencing personalized rates of health span.

Recent research has highlighted the significant role of lncRNAs in regulating muscle regeneration. Among these lncRNAs, PAM (Pax7 Associated Muscle lncRNA), is activated in satellite cells (SCs) following injury, driving their differentiation into myoblasts. PAM originates from a muscle-specific super-enhancer and functions, predominantly affecting Ddx expression in trans (So et al., 2022). Interestingly, aged MuSCs exhibit increased PAM expression, suggesting its involvement in age-related muscle alterations. In the study by Cai et al. (2019), a novel lncRNA called Lnc-ORA was identified as being associated with obesity. Lnc-ORA was found to increase in middle-aged muscle (52 weeks old) and during myogenic differentiation, promoting muscle proliferation while suppressing differentiation (Cai et al., 2021). Treatment with dexamethasone increased lnc-ORA expression, leading to muscle atrophy. Lnc-ORA predominantly acted as a ceRNA against miR-532-3p, ultimately modulating the Akt and mTOR pathway and key myogenic transcriptional regulators, including MyoD and MyHC. Four molecular markers, GOT1, SEPP1, SV2A, and GFOD1, known to prevent muscle differentiation and regeneration were used by Wang et al. to develop a novel method for predicting sarcopenia (Wang et al., 2020). Their research also unveiled the role of lncRNA DLEU2 as a miR-181a sponge, effectively suppressing skeletal muscle regeneration and differentiation. Furthermore, the lncDLEU2-miR-181a-SEPP1 pathway emerges as a promising therapeutic target for mitigating age-related sarcopenia by modulating muscle differentiation and regeneration. Another lncRNA 2310043L19Rik, also known as lnc-231, was found to be highly expressed in muscle, with its expression decreasing in aged muscle (Li et al., 2020). Functional analysis revealed that the overexpression of lnc-231 restricted myoblast differentiation while promoting their proliferation *in vitro*. Conversely, inhibiting lnc-231 had the opposite effect. Lnc-231 targeted miR-125a-5p by acting as a competitive endogenous RNA (ceRNA), whereas miR-125a-5p binds to the 3′UTR region of the E2F3 mRNA to inhibit it. Collectively, lnc-231 plays a role in promoting myoblast proliferation while inhibiting differentiation by modulating the activity of miR-125a-5p. Recent research has highlighted the upregulation of a lncRNA called cytoskeletal regulator (CYTOR) in both human and rodent skeletal muscle following various exercise regimens (Wohlwend et al., 2021). Conversely, CYTOR exhibited a decline with age, which was associated with the attenuation of muscle weight loss, enhanced muscle strength, and an increase in the proportion of fast-type muscle fibers. Particularly fascinating were the findings demonstrating that overexpression of CYTOR in aged human myoblasts improved the capability for myogenic differentiation and boosted the production of fast-twitch myosin isoforms. Taken together, these investigations provide light on the crucial functions of lncRNAs in muscle biology, providing valuable insights into the intricate processes of muscle regeneration and aging. These lncRNAs characterized in muscle aging were summarized in Table 2.

**TABLE 2:** Characterization of lncRNA in muscle aging.

lncRNA	Expression^a^	Target genes	Biological function	Reference
AC004797.1, PRKG1-AS1 and GRPC5D-AS1	↑	?	Knockdown of PRKG1-AS1 increases cell viability, decreases cell apoptosis, and upregulates the expression of MyoD, MyoG, and Mef2c	Zheng et al. (2021)
Synaptopodin-2	↑	miR-23a-3p miR-103-3p miR-205-5p	Synaptopodin-2 increases the expression of FOXO3a, Atrogin-1 and MuRF1 by acting as a sponge for miRNAs	Jin et al. (2018)
lncMAAT	↓	miR-29b Sox6 Mbnl1	LncMAAT alleviated muscle atrophy *in vitro* suppressed Atrogin-1 and MuRF1	Li et al. (2021)
PAM	↑	Ddx5	PAM leads to enhanced inter-chromosomal interactions and activates Timp2 and Vim	So et al. (2022)
lnc-ORA	↑	miR-532-3p	Lnc-ORA promotes cell proliferation and while suppressing differentiation through activation of Akt/mTOR pathway	Cai et al. (2021)
lncDLEU2	↑	miR-181a SEPP1	LncDLEU2 suppressed skeletal muscle regeneration	Wang et al. (2020)
lnc-231	↓	miR-125a-5p E2F3	Lnc-231 promotes myoblast differentiation	Li et al. (2020)
CYTOR	↓	Tead1	CYTOR attenuates muscle loss and enhances muscle strength by increasing the proportion of fast-type muscle fibers	Wohlwend et al. (2021)

^a^ The arrows indicating the expression of lncRNAs, in aged muscle or serum, comparing to young control, was as follows.

### 2.3 circRNAs in muscle aging

CircRNAs are a subclass of non-coding RNA molecules that form covalently closed continuous loops (Sanger et al., 1976). CircRNAs originate from canonical splice sites and are generated through processes like linear splicing or back splicing during transcription (Kristensen et al., 2019). These circular structures make circRNAs highly stable and resistant to degradation by cellular machinery, contributing to their longevity within cells (Enuka et al., 2016). They play diverse roles, including acting as microRNA sponges that sequester and modulate the activity of microRNAs (Memczak et al., 2013). Furthermore, circRNAs engage in interactions with RNA-binding proteins, exerting significant influence over gene expression and participating in post-transcriptional regulatory mechanisms.

The role of circRNAs in muscle aging is an increasingly prominent topic in current research due to their multifaceted involvement in the complex processes of muscle deterioration with age (Abdelmohsen et al., 2015; Mahmoudi and Cairns, 2019). Recently, it was reported that circBBS9 and circRNA FUT10 are differentially expressed in aging muscles (Guo et al., 2020; Zhu et al., 2021). CircBBS9 showed lower levels in older muscles compared to younger ones and higher expression in response to exercise compared to resting states. Mechanistically, circBBS9 was found to participate in a circBBS9-miRNA-mRNA network, suggesting that it might actively contribute to muscle aging by enhancing the effects of programs involving aerobic training. Importantly, overexpression of circBBS9 led to a decrease in muscle atrophy-related genes and an increase in functional mitochondrial genes, such as Mfn1, Pgc1α, and Atpase, in differentiated C2C12 myotubes. Collectively, these results imply that circBBS9 overexpression may contribute to muscle aging and potentially enhance gene programs that control muscle functionality. In aged MuSCs, circRNA FUT10 was identified to be upregulated compared to adult MuSCs. Furthermore, circRNA FUT10 suppresses MuSCs proliferation and differentiation as a sponge to bind miR-365a-3p and the 3′UTR of HOXA9 mRNA. These findings give important new understandings into the mechanics of MuSC aging and suggest a possible therapeutic target for the treatment of degenerative muscle diseases. These circRNAs characterized in muscle aging were summarized in Table 3.

**TABLE 3:** Characterization of circRNA in Muscle Aging.

circRNA	Expression^a^	Target genes	Biological function	Reference
circBBS9	↓	?	Overexpressed circBBS9 inhibits muscle atrophy genes and activates mitochondrial related genes	Guo et al. (2020)
circFUT10	↑	miR-365a-3p HOXA9	circFUT10 suppresses MuSC proliferation and differentiation	Zhu et al. (2021)

^a^ The arrows indicating the expression of circRNAs, in aged muscle or serum, comparing to young control, was as follows.

## 3 ncRNAs as potential therapeutic targets for delaying muscle aging

Non-coding RNAs have emerged as a fascinating and promising frontier in the field of molecular biology and therapeutics (Winkle et al., 2021). Previously disregarded as junk DNA, these ncRNAs are now understood to have a crucial function in controlling gene expression and directing complex cellular processes. Notably, miRNAs and lncRNAs have garnered substantial attention due to their ability to fine-tune gene expression patterns and modulate various biological pathways. The therapeutic potential of ncRNAs lies in their capacity to act as molecular switches, either by silencing harmful genes or by activating beneficial ones. Researchers are exploring the use of synthetic ncRNAs, RNA-based drugs, and gene-editing technologies to target ncRNAs for treating several diseases including cancer (Wang et al., 2019), neurodegenerative disorders (Salvatori et al., 2020), and cardiovascular diseases (Huang et al., 2020). Their ability to target previously undruggable genes make these therapeutics a promising tool in the ongoing pursuit of more effective and personalized treatments for a wide array of medical conditions.

Non-coding RNA therapeutics might be a promising strategy in the quest to delay muscle aging (Liu et al., 2021b). One class of ncRNAs, miRNAs, has gained significant attention for their ability to regulate key molecular pathways involved in muscle maintenance and regeneration. They can negatively regulate the expression of genes involved in muscle protein synthesis and satellite cell activation. Targeting these miRNAs with antisense oligonucleotides or miRNA mimics holds promise for restoring muscle function and combating age-related muscle decline. In addition, lncRNAs are known to modulate the activity of muscle-specific genes and influence muscle differentiation and regeneration. By designing therapies that target these specific lncRNAs, researchers aim to rejuvenate aged muscles and enhance their regenerative capacity. The development of RNA-based therapeutics, such as CRISPR-Cas9-mediated gene editing or RNA interference (Li et al., 2023), offers exciting opportunities to precisely manipulate the expression of these ncRNAs and potentially reverse age-related muscle wasting. Furthermore, circRNAs have recently emerged as potential players in muscle aging, with studies uncovering their roles in regulating muscle-specific genes and cellular processes. Manipulating circRNAs could provide novel avenues for therapeutic intervention.

Exploring the intricate role of ncRNAs in muscle aging has ignited excitement for potential therapeutic breakthroughs. However, this emerging field also presents a series of challenges that demand meticulous attention for the full realization of ncRNAs as therapeutic targets in the context of muscle aging. One of the central challenges revolves around deciphering the complex regulatory networks orchestrated by ncRNAs in muscle aging. These networks exhibit a high degree of complexity, encompassing various types of ncRNAs, including miRNAs, lncRNAs, and circRNAs. Thus, gaining a comprehensive understanding of their respective roles and intricate cross-talk within these networks is imperative. The identification of key nodes in these networks and an understanding of their functional significance are crucial steps in the development of targeted interventions. Moreover, achieving the therapeutic potential of ncRNAs in muscle aging hinges on minimizing unintended side effects and off-target consequences. Precision is paramount to realizing the desired therapeutic outcomes, underscoring the importance of addressing and mitigating potential off-target effects. Another challenge pertains to the delivery of ncRNA-based therapeutics to muscle tissues effectively. Developing efficient delivery systems that can specifically target muscle cells while avoiding off-target effects is a priority. Many researchers have focused their efforts toward the development of adeno-associated virus-based systems for ncRNA delivery, a solution that offers not just higher stability but also increased efficiency over oligonucleotides (Winkle et al., 2021). Furthermore, the viral system’s use of muscle-specific promoter sequences, such as skeletal muscle α-actin, muscle creatine kinase, and desmin, helps get around impediments in the way of whole-body muscle-specific targeting and prevent off-target effects (Skopenkova et al., 2021). Overcoming barriers related to delivery, stability, and bioavailability of ncRNAs remains an ongoing challenge in the field.

## 4 Conclusion

**FIGURE 1:**
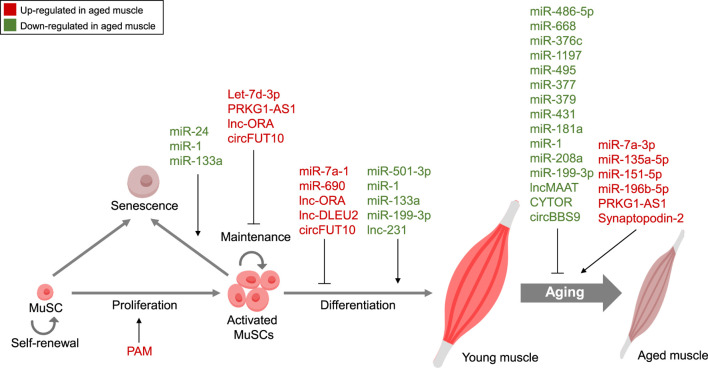
Non-coding RNA-Mediated Regulation in Muscle Aging. Summary of ncRNAs, including miRNAs, lncRNAs, and circRNAs, controlling diverse features of muscle aging. *Red*, Upregulated ncRNAs in aged muscle; *Green*, Downregulated ncRNAs in aged muscle.

In recent years, research into ncRNAs and their role in muscle aging has gained significant momentum, reflecting a burgeoning trend in the field of gerontology and molecular biology. The exploration of miRNAs, lncRNAs, and circRNAs has unveiled a complex web of regulatory networks that govern various aspects of muscle aging, summarized in Figure 1. Researchers have been fervently identifying specific ncRNAs associated with age-related muscle decline, elucidating their mechanisms of action, and deciphering their impact on processes like muscle regeneration, protein synthesis, and inflammation. Our knowledge of the molecular causes of muscle aging has been expanded by improvements in high-throughput sequencing technology and bioinformatics. These advancements have accelerated the discovery of novel ncRNAs and their targets. The therapeutic potential of ncRNAs as targets for delaying muscle aging is a promising and rapidly evolving field, despite challenges exist. By overcoming these challenges and harnessing the intricate regulatory functions of these molecules, researchers are striving to develop innovative treatments that may help individuals maintain muscle health and function as they age, ultimately improving the quality of life for aging populations.
